# Phase Change Microcapsule Composite Material with Intelligent Thermoregulation Function for Infrared Camouflage

**DOI:** 10.3390/polym15143055

**Published:** 2023-07-15

**Authors:** Ying Su, Xiaoming Zhao, Yue Han

**Affiliations:** 1 School of Textile Science and Engineering, Tiangong University, Tianjin 300387, China; 2 Tianjin Key Laboratory of Advanced Textile Composites, Tiangong University, Tianjin 300387, China; 3 Tianjin Municipal Key Laboratory of Advanced Fiber and Energy Storage, Tiangong University, Tianjin 300387, China

**Keywords:** phase change microcapsules, infrared camouflage, emissivity, intelligent temperature regulation, textile composites

## Abstract

The infrared camouflage textile materials with soft and wear-resistant properties can effectively reduce the possibility of soldiers and military equipment being exposed to infrared detectors. In this paper, the infrared camouflage textile composites with intelligent temperature adjustment ability were prepared by different methods, using phase change microcapsule as the main raw material and high polymer polyurethane as the matrix, combining the two factors of temperature control and emissivity reduction. It was tested by differential scanning calorimeter, temperature change tester, infrared emissivity tester, and infrared imager. The results show that the temperature regulation effect of textile materials finished by coating method is better than dip rolling method, the temperature regulation ability and presentation effect are the best when the microcapsule content is 27%. When the bottom layer of infrared camouflage textile composite is 27% phase change microcapsule and the surface layer is 20% copper powder, its infrared emissivity in the band of 2–22 μm is 0.656, and the rate of heating and cooling is obviously slowed down. It has excellent heat storage and temperature regulation function, which can reduce the skin surface temperature by more than 6 °C and effectively reduce the infrared radiation. This study can provide reference for laboratory preparation and industrial production of infrared camouflage composite material. The infrared camouflage textile composite prepared are expected to be used in the field of military textiles.

## 1. Introduction

Matter is generally divided into three phases: solid, liquid, and gas. The transition between different phase states of the same material is called phase transition [1]. The substance whose state can be changed is called phase change material (PCM). When the phase change occurs, there is a significant energy exchange between the material and the environment, which will be strongly coupled with the heat transfer, so that the material has a certain temperature control and heat release function [2,3]. With this capability of phase change materials, the temperature around the working source or materials can be adjusted and controlled to reduce the mismatch between energy supply and demand in time and speed [4]. Therefore, phase change materials are applied broadly in the field of energy storage and temperature regulation [5,6]. However, phase change materials have problems such as large volume changes, easy leakage, and low thermal conductivity. Microencapsulation of phase change materials is an advanced application method. Microcapsule phase change material (MPCM), also known as phase change microcapsule, is a new type of composite phase change material with core-shell structure. It is coated with a stable polymer film on the surface of solid-liquid phase change material particles. The shell structure of microcapsules can provide good protection for phase change core materials, improve the stability of phase change materials, prevent chemical reactions with the outside world and leakage during long-term cyclic use, and significantly increase the contact area with the matrix material to improve thermal conductivity, thereby improving the working performance of phase change materials [7,8,9]. When the external temperature changes, the core material in the microcapsule will undergo phase change. The phase change material absorbs or releases a large amount of heat, and the temperature of the microcapsule itself remains constant, to achieve the effect of intelligent temperature regulation [10,11,12,13]. Phase change microcapsules with temperature regulation ability are widely used in construction [14,15,16,17,18], solar energy [19], food industry [20], textile [21,22], and other fields.

In the modern battlefield, the infrared radiation energy of general military targets is higher than the background, so it is easy to find out by using infrared detectors. According to Stefan Boltzmann’s Law (1) [23], the total infrared radiation energy of an object is directly proportional to the fourth power of its emissivity and absolute temperature. Therefore, the possibility of the target being discovered by the infrared detector can be reduced by reducing the emissivity and controlling the temperature, to achieve its camouflage effect in the infrared band. Therefore, infrared camouflage materials that protect military targets without changing the shape and structure of the target have attracted extensive attention in the national defense and military industry [24]. Various infrared camouflage materials developed around fibers and fiber products are called infrared camouflage textile materials. Infrared camouflage textile materials are soft, portable, and wearable. They are the main raw materials of infrared camouflage clothing, backpacks, camouflage nets and tents. They can provide guarantee for the survival of soldiers and weapons and equipment, and plays an extremely important role in the battlefield [25,26]. In terms of reducing infrared emissivity, it mainly includes developing new low emissivity fibers [27,28,29,30], modifying existing fibers [31,32,33] or coating low infrared emissivity coatings [34,35,36]. In terms of temperature control, in addition to thermal insulation and structural design [37,38,39,40], the temperature regulating textile [41,42,43,44] and infrared camouflage textile [45,46,47] are prepared by combining phase change microcapsules with textile, which can effectively reduce the infrared radiation energy of the target [48,49,50]. However, it is difficult to use phase change microcapsules for infrared camouflage alone. Its phase change temperature, latent heat of phase change, and thermal conductivity can hardly meet the requirements of thermal camouflage. Only by combining with other functional materials can infrared camouflage be better realized [51]. According to Kirchhoff’s law, opaque objects with high reflectivity generally have low emissivity. Metal is a typical low-emissivity material, which is generally used in the field of infrared camouflage in the form of coating. Among them, copper and aluminum have become the main force of metal fillers due to their low cost and easy availability, excellent performance, and wide application. In this paper, phase change microcapsules are finished on the fabric, and the temperature-regulated fabric is obtained by changing different parameters. On this basis, the infrared camouflage fabric was prepared by adding low emissivity materials. Then its temperature adjustment ability, infrared camouflage effect, and mechanism are analyzed systematically. Compared with the untreated fabric, the prepared textile has a certain degree of infrared camouflage ability, which can delay the speed of temperature rise and effectively reduce the infrared thermal radiation.

*E* = *σεT*^4^ (1)

where *E* is the infrared radiation (J/(s·m^2^)), *σ* is Stefan Boltzmann constant, *ε* is the emissivity of the target surface, and *T* is the thermodynamic temperature of the target surface (K).

## 2. Materials and Methods

### 2.1. Materials

The fabric used in the experiment was cotton fabric, which was purchased from Hongfei Textile Manufacturing, Baoding, China. Phase change microcapsules are prepared by in-situ polymerization with paraffin as core material and urea-formaldehyde resin as wall material. Urea and formaldehyde aqueous solutions were purchased from Meryer (Shanghai) Chemical Technology, Shanghai, China. Paraffin, OP emulsifier, triethanolamine, citric acid and petroleum ether were purchased from Beijing enokai Technology, Beijing, China. Polyurethane resin was purchased from Guangzhou Yuheng environmental protection materials, China. Hollow glass beads were purchased from Henan Bairun casting materials, Zhengzhou, China. Silicon dioxide was purchased from Jiangsu Tianxing’s new materials, Huaian, China. The copper powder was purchased from Nangong Xindun alloy welding material spraying, Xingtai, China. Aluminum powder was purchased from Shanghai Aladdin Biochemical Technology, Shanghai, China. All reagents were of analytical grade and used directly without further purification. The details are shown in Table 1.

### 2.2. Methods

#### 2.2.1. Preparation of Phase Change Microcapsules

Mix urea and formaldehyde aqueous solution in a certain proportion, drop triethanolamine to adjust the pH value of the solution to be weakly alkaline, react at 70 °C for 1 h, and add deionized water to form a stable urea/formaldehyde prepolymer solution. Add a certain amount of OP emulsifier and paraffin into deionized water, heat and melt, emulsify and disperse for 30 min (3000 r/min) at 60 °C, and form a stable emulsion. Drop the prepolymer solution into the emulsion and stir for 20 min after dropping. Then slowly add citric acid solution, adjust the final pH value of the solution to be acidic, keep the temperature at 60 °C for reaction for 1 h, and then raise the temperature to 90 °C, and keep the temperature for reaction for 2 h. After the reaction, the microcapsule lotion was poured out, cooled, separated and filtered. The obtained microcapsules were washed twice with petroleum ether and deionized water, and dried to obtain white powder microcapsules.

#### 2.2.2. Preparation of Phase Change Microcapsule Temperature Regulating Textile by Dip Rolling

Disperse the phase change microcapsule particles in water, add dispersant, adhesive, and penetrant, mix evenly to obtain the phase change microcapsule solution. The cotton fabric with 10 × 10 cm^2^ was washed and dried. Put it into a beaker containing phase change microcapsule solution and fully wet it, with a bath ratio of 1:20. The phase change microcapsule fabric was obtained by two dipping and two rolling processes, drying at 80 °C for 5 min, and then baking at 120 °C for 2 min. Two groups of phase change microcapsule fabrics were prepared by changing the microcapsule content and adhesive content respectively.

#### 2.2.3. Preparation of Phase Change Microcapsule Temperature Regulating Textile by Coating Method

Disperse the phase change microcapsule particles in water, add adhesive, thickener, dispersant and defoamer, and stir evenly to obtain phase change microcapsule coating. Take 20 × 20 cm^2^ cotton fabric, washed, dried, and ironed flat. The coating was evenly coated on the cotton fabric by a small sample coating machine, dried at 80 °C for 10 min, and dried to obtain the phase change microcapsule fabric. Three groups of phase change microcapsule fabrics were prepared by changing the content of phase change microcapsule, the thickness of coating and the type of thermally conductive materials.

#### 2.2.4. Preparation of Infrared Camouflage Textile

In this paper, aluminum powder and copper powder are selected as coating materials with low emissivity, combined with phase change microcapsules to achieve a better-infrared camouflage effect on the fabric. Firstly, aluminum powder or copper powder is directly added into the phase change microcapsule solution, mixed evenly to obtain the infrared camouflage coating, and according to Section 2.2.3 to prepare infrared camouflage fabric. In addition, aluminum powder or copper powder shall be directly mixed with the adhesive to obtain low emissivity coating, and then phase change microcapsule coating can be obtained according to Section 2.2.3. The infrared camouflage fabric was prepared by coating phase change microcapsule coating and low emissivity coating on cotton fabric in turn.

### 2.3. Characterizations and Measurements

The characteristic functional groups of samples were measured by infrared spectrometer (FRONTIER, FT-IR, made by PerkinElmer, Waltham, MA, USA). The phase change microcapsules and fabrics were observed by scanning electron microscope (HITACHI, SEM, made by Hitachi Limited, Tokyo, Japan), and the distribution of microcapsules and metal particles on the fabric surface was compared. The enthalpy values of phase change microcapsules, unfinished fabrics and phase change microcapsule temperature regulating fabrics were measured by differential scanning calorimetry (NETZSCH, DSC200F3, made by Netzsch Group, Bavaria, Germany). The heating temperature range was 20–50 °C, the cooling temperature range was 50–20 °C, and the heating and cooling rates were 5K/min. The time when the sample rises to a specific temperature is measured by a self-made temperature rising instrument to evaluate its temperature regulation ability. The infrared thermal source is an infrared lamp (Philips 175R, made by Philips, Amsterdam, The Netherlands). The temperature rising range is 20–60 °C. The unfinished fabrics and infrared camouflage fabrics with different parameters were heated on a constant temperature (simulating human body surface temperature) test bench, and the infrared thermal image was taken with an infrared thermal imager (FlIR^®^TG165, made by FLIR Systems, Wilsonville, OR, USA) to test their thermal insulation and infrared camouflage properties. The emissivity of infrared camouflage fabric was measured by far infrared emissivity tester (TSS-5X, made by Japan sensor corporation, Tokyo, Japan).

## 3. Results

### 3.1. Characterization and Analysis of Phase Change Microcapsules

**Figure 1:**
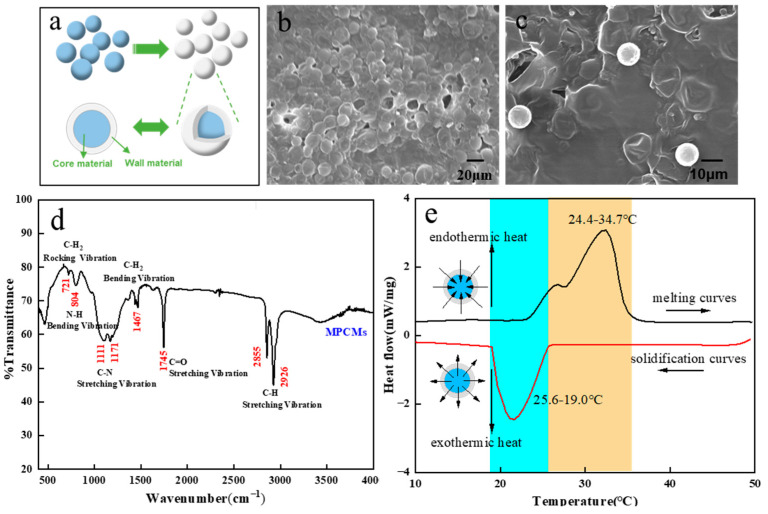
(**a**) Structure diagram of phase change microcapsule, (**b**,**c**) SEM of phase change microcapsules under different magnification of electron microscope, (**d**) Fourier transform infrared spectroscopy of phase change microcapsules, (**e**) DSC curve of phase change microcapsules.

The phase change microcapsules used in this experiment are prepared with paraffin as the core material and urea formaldehyde resin as the wall material. The structural diagram is shown in Figure 1a. From the SEM Figure 1b,c, it can be seen that the phase change microcapsule is spherical with a particle size of 10 μm or so. According to the infrared spectrum in Figure 1d, the phase change microcapsule has 7 absorption peaks. The absorption peaks at 2926 cm^−1^ and 2855 cm^−1^ are related to the asymmetric and symmetric stretching vibration of C-H, the absorption peak at 1467 cm^−1^ is caused by the bending vibration of C-H_2_, the absorption peak at 721 cm^−1^ is caused by the rocking vibration of C-H_2_, and the absorption peak at 1745 cm^−1^ is caused by the stretching vibration of C=O, the absorption peaks at 1171 cm^−1^ and 1111 cm^−1^ are caused by the stretching vibration of C-N, and the band at 804 cm^−1^ corresponds to the bending vibration of N-H. It can be seen that there are both characteristic peaks of paraffin and urea formaldehyde resin in the infrared spectrum, which also proves that the microcapsule is composed of wall urea formaldehyde resin and core paraffin. Figure 1e shows the DSC curve of phase change microcapsules. The heat storage and temperature regulation performance of phase change microcapsules are mainly determined by the solid-liquid phase change of its core paraffin. During heating up, the phase change microcapsules began to melt and absorb heat at 24.4 °C, and the phase change temperature range was 24.4–34.7 °C. During cooling, the phase change microcapsules began to solidify and release heat at 25.6 °C, and the phase change temperature range was 25.6–19.0 °C. According to the indicators formulated by outlast, the body surface contact air layer is 18.3–29.4 °C, belonging to the cold climate temperature area, the body surface contact air layer is 26.7–37.8 °C, belonging to the mild or comfortable temperature area, and the body surface contact air layer is 32.2–43.3 °C, belonging to the temperature area during hot or intense exercise [52]. The phase change microcapsule used in this paper can be combined with fabric to adjust the temperature and infrared radiation energy of the body surface air layer in cold climates.

### 3.2. Preparation and Performance Analysis of Phase Change Microcapsule Temperature Regulating Fabric

**Figure 2:**
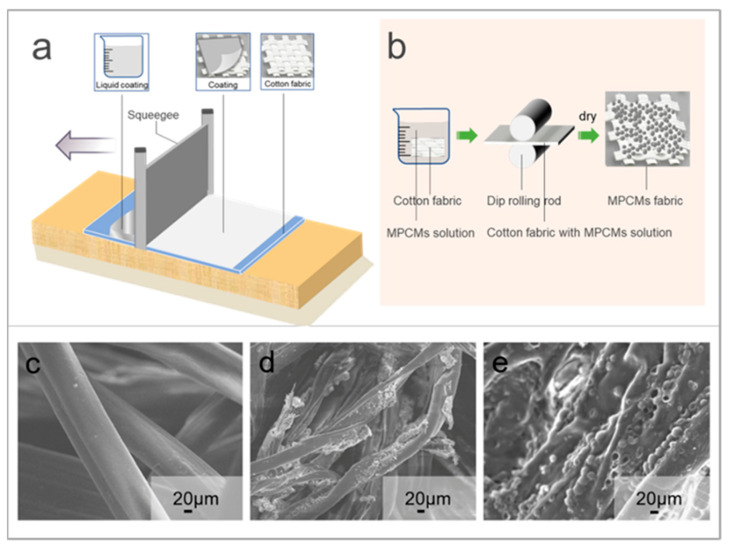
(**a**) Preparation of phase change microcapsule temperature control fabric by coating method, (**b**) Preparation of phase change microcapsule temperature control fabric by dip rolling, (**c**) SEM image of untreated cotton fabric, (**d**) SEM image of samples prepared by dip rolling method, (**e**) SEM image of samples prepared by coating method.

According to the steps described in Section 2.2.2 and Section 2.2.3, phase change microcapsules are combined with cotton fabric by dip rolling and coating (Figure 2a,b). Before finishing, the surface of cotton fiber is smooth and tidy, flat and longitudinally twisted (Figure 2c). The phase change microcapsule solution was treated on the cotton fabric by dip rolling. The observation of the SEM (Figure 2d) showed that the phase change microcapsule particles were attached to the bending and depression of the cotton fiber through the adhesive. The phase change microcapsule coating was applied to the cotton fabric. It can be seen from the SEM image (Figure 2e) that the adhesive wrapped the phase change microcapsule and covered the surface of the cotton fabric to form a complete coating.

**Figure 3:**
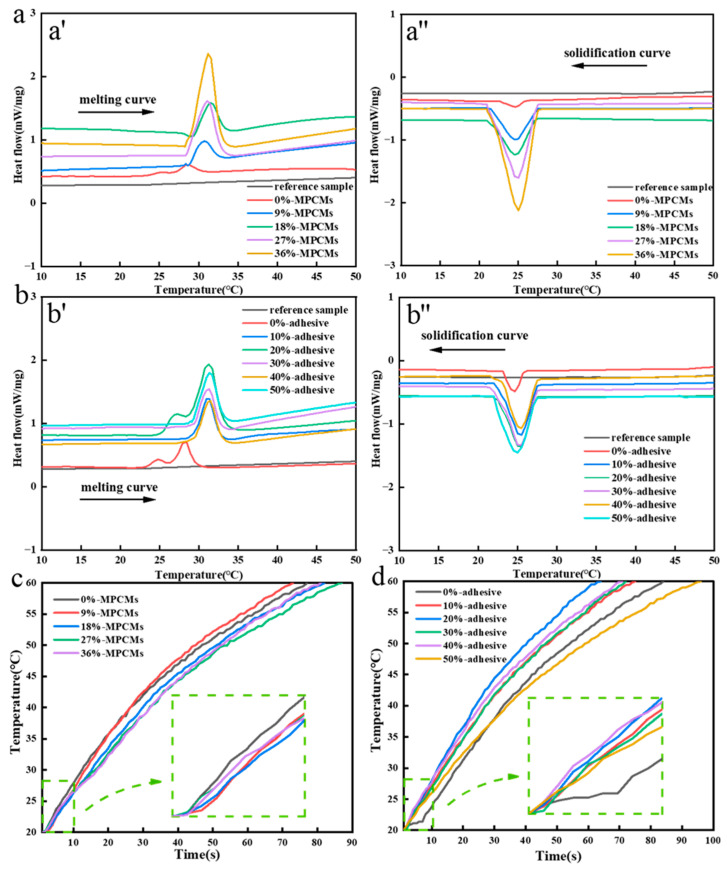
Dip rolling method (**a**) DSC curve of samples with different content of phase change microcapsules, (**b**) DSC curve of samples with different binder content, (**c**) Temperature rise curve of samples with different content of phase change microcapsules, (**d**) Temperature rise curve of samples with different binder content.

When the phase change microcapsule temperature regulating fabric is prepared by dip rolling, the process parameters shall be consistent, the content of phase change microcapsules and adhesives is the main factor affecting the related properties of fabrics. Therefore, we analyzed the effects of different content of phase change microcapsules and adhesives on the temperature regulation ability of the fabric. The reference sample in the figure is untreated cotton fabric. It can be seen from Figure 3a that the content of phase change microcapsules basically does not affect the phase change initial temperature of the sample. The initial temperature of exothermic and endothermic is around 28 °C. The latent heat of phase transformation of the sample increases with the increase of the content of microcapsules, and the latent heat of phase transformation of the sample is the largest when the percentage content is 36%, because the more the content, the more phase change microcapsules attached to the fabric after dip rolling treatment, and the greater the overall latent heat of phase change. The fabric treated by phase change microcapsule can absorb or release a certain amount of heat, so as to achieve the purpose of temperature regulation. As can be seen from Figure 3b, the phase transition latent heat of the sample increases with the increase of the binder content. Because the larger the binder content, the more phase change microcapsules adhere to the fabric surface. The greater the latent heat of phase change of fabric, the more obvious the effect of heat absorption and release. With the increase of binder content, the phase transition temperature of the sample will also change. The reason is that the added adhesive can block the heat transfer and increase the phase transition temperature of the sample. Figure 3c shows the temperature rise curve of samples with different content of phase change microcapsules. When the content of the phase change microcapsule is 9%, the heating rate of the sample is the fastest, and it takes 65 s to rise from 20 to 60 °C. When the content of the phase change microcapsule is 27%, the heating rate of the sample is the slowest, and it takes 83 s to rise from 20 to 60 °C. That is when the content of phase change microcapsule is 27%, the temperature regulation and heat storage effect of the sample is the best. The reason is that the existence of phase change microcapsules will delay the rate of fabric temperature change, that is, the heat released and absorbed will be temporarily supplemented and stored through phase change materials. When the content of phase change microcapsules is 36%, the heating rate of the sample is not the slowest. The reason is that when the content of phase change microcapsules in the solution is too high, agglomeration will occur in the mixing process, resulting in poor dispersion effect of phase change microcapsules, which affects the overall heat storage capacity of the sample. Figure 3d is the temperature rise curve of the sample with different adhesive content. When the adhesive content is 50%, the heating rate of the sample is the slowest, and the time is 95 s. When the content of adhesive is 20%, the heating rate of the sample is the fastest, and the time is 60 s. This may be because when the binder content is low, the content of phase change microcapsules entering the fabric interior and attached to the fabric surface after dip rolling treatment is relatively small. When the binder content is high, the proportion of the binder solidified on the fabric surface will increase, and a layer of film will be formed on the fabric surface, which will inhibit the heat transfer and slow down the heating rate of the sample. At the same time, the high content of adhesive will also increase the phase change microcapsules adhered to the fabric surface and immersed into the fabric, and further reduce the heating rate of the sample.

When the phase change microcapsule temperature regulating fabric is prepared by coating method, the process parameters shall be consistent, the content of phase change microcapsules and coating thickness are the main factors affecting the related properties of fabrics, and the thermal conductivity also has a great influence on the temperature regulation ability of materials. Therefore, we analyzed the effects of phase change microcapsule content, coating thickness and thermal conductivity on the temperature regulation ability of the fabric. The reference sample in the figure is untreated cotton fabric.

**Figure 4:**
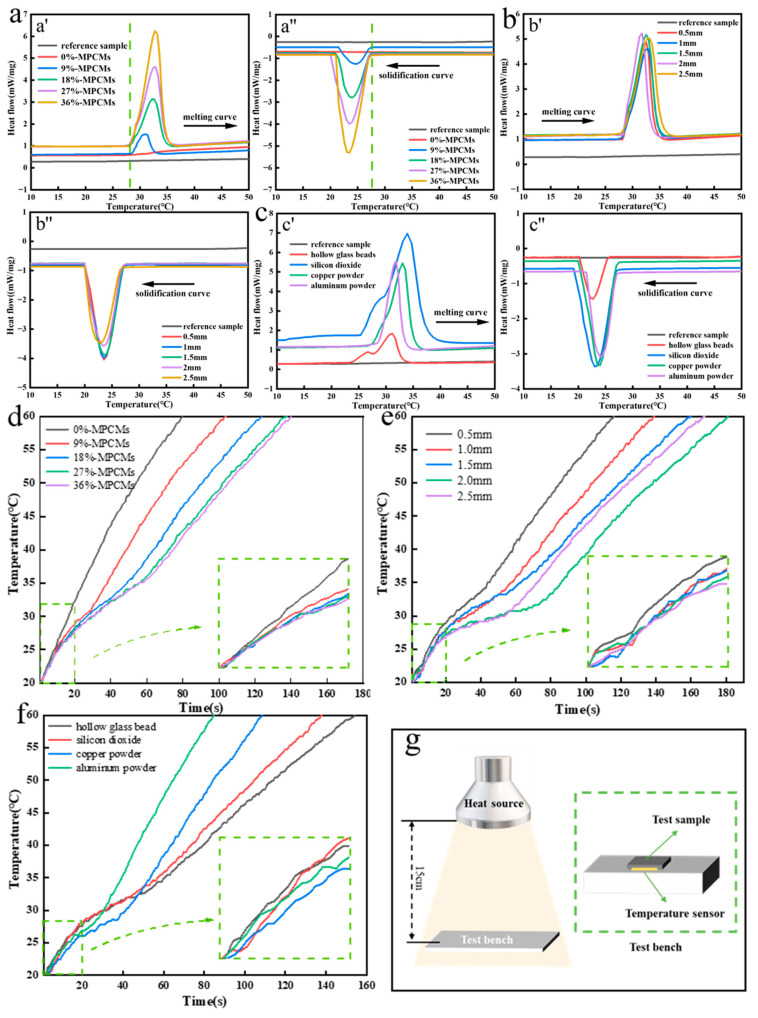
Coating method (**a**) DSC curve of samples with different content of phase change microcapsules, (**b**) DSC curves of samples with different coating thickness, (**c**) DSC curves of samples with different thermal conductivity, (**d**) Temperature rise curve of samples with different content of phase change microcapsules, (**e**) Temperature rise curve of samples with different coating thickness, (**f**) Temperature rise curve of samples with different thermal conductivity, (**g**) Schematic diagram of heating rate test.

Figure 4a,c show the DSC curves of samples with different content of phase change microcapsule, different coating thickness and different thermal conductivity materials. It can be seen from Figure 4a that the adhesives and other additives have no influence on the phase change temperature and latent heat of the phase change microcapsule fabric. With the increase of the content of phase change microcapsules in the coating, more and more microcapsule phase change materials are attached to the surface of the fabric, which improves the heat storage and temperature adjustment ability of the prepared sample. However, in the experiment, it is found that when the content of phase change microcapsules in the coating is too high, the phenomenon of agglomeration and uneven dispersion will appear, and cracks will appear on the surface of the prepared coating. Therefore, the content of phase change microcapsules in the coating was fixed at 27% in the subsequent experimental study. It can be seen from Figure 4b that the coating thickness also has a certain effect on the phase transformation latent heat of the sample. When the phase change microcapsules melt endothermically, the phase change latent heat of the samples with coating thickness of 1.5 mm and 2.0 mm is close and relatively large. When the phase change microcapsules solidify exothermically, the phase change latent heat of the samples with coating thicknesses of 1.0 mm and 1.5 mm is close and relatively large. There is no positive correlation between thickness and latent heat of phase change. The main reason is that the sample taken in the DSC test is very small, and the larger the thickness, the smaller the sampling area, which cannot completely guarantee the content of dispersed phase change microcapsules in the test sample. Considering comprehensively, in the subsequent experimental research, the thickness of the coating is determined as 1.5 mm. When the coating thickness is 1.5 mm and the content of phase change microcapsules is 27%, the addition of materials with different thermal conductivity will also have a great influence on the heat storage and temperature adjustment ability of the sample. It can be seen from Figure 4c that adding different materials to the coating will have a certain influence on the initial temperature of the phase transition of the sample. When adding hollow glass beads and silicon dioxide thermal insulation materials with low thermal conductivity, the phase transition temperature of the sample will be reduced. When copper powder and aluminum powder with high thermal conductivity are added, the phase change latent heat of the sample is similar. The latent heat of melting phase transformation of samples containing silicon dioxide is the largest. This is because the silicon dioxide used in the experiment is nano-scale, and has the advantages of large specific surface area and high porosity. It can absorb a certain calorific value when heating up.

Figure 4d–f show the temperature rise curves of samples prepared by coating method with different content of phase change microcapsules, different coating thicknesses, and different thermal conductivity materials. It can be seen from the figure that the higher the content of phase change microcapsules, the longer the time required for the sample to rise from 20 to 60 °C, and the heating rate of the sample before 30 °C is slow. When one side of the fabric is heated, most of the heat is absorbed by the phase change microcapsules in the process of transferring the heat radiation to the other side of the fabric. Therefore, the higher the content of the phase change microcapsule, the more heat the sample and the slower the heating rate. When the coating thickness is 2.0 mm, the heating rate of the sample is the slowest, and the time for the sample to rise from 20 to 60 °C is 180 s. Materials with different thermal conductivity are added to the coating. When the materials are hollow glass beads and silicon dioxide, the heating speed of the sample is slow because of its low thermal conductivity. The sample with hollow glass beads has the slowest heating rate, mainly because the hollow structure of hollow glass beads is closed and contains more still air, which further enhances its thermal insulation performance. Figure 4g is the schematic diagram of sample heating rate test. To compare the thermal insulation capacity of the sample in a short time, the distance between the light source and the sample is close, so the heating speed will be much faster than in the actual situation.

**Figure 5:**
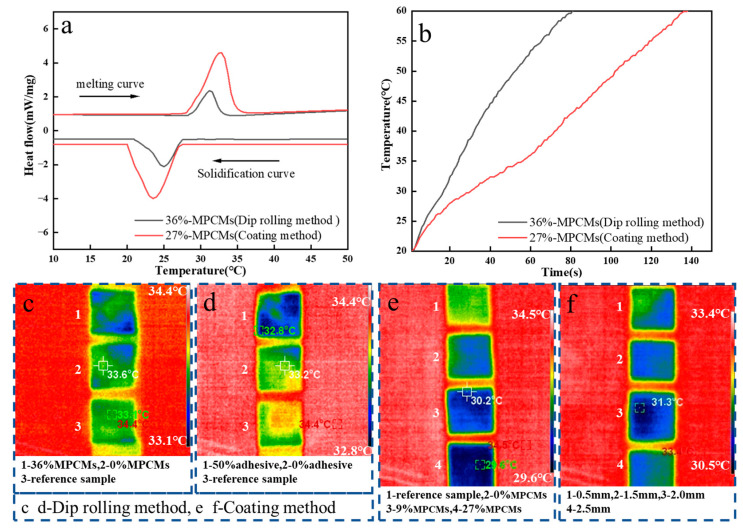
(**a**) Comparison of DSC curves between samples selected from dip rolling method and coating method, (**b**) Comparison of temperature rise curves between samples selected from dip rolling method and coating method, (**c**) Infrared thermogram of samples with different content of phase change microcapsules, (**d**) Infrared thermogram of samples with different binder content, (**e**) Infrared thermogram of samples with different content of phase change microcapsules, (**f**) Infrared thermogram of samples with different coating thickness.

Select the best samples prepared by the dip rolling method and coating method to compare and analyze. The experimental results show that, compared with the dip rolling method, the temperature-regulating fabric with phase change microcapsules prepared by the coating method has a greater latent heat of phase change (Figure 5a) and better temperature control ability (Figure 5b). Because the amount of phase change microcapsules on the sample in the coating method is more and the adhesion is firmer. Through the observation of the sample by the infrared thermal imager, it is also confirmed that the sample prepared by the coating method has better infrared camouflage effect (Figure 5c–f). Considering the performance, cost, thickness and other factors, we think that the content of phase change microcapsules is 27% and the coating thickness is 1.5 mm, which is the best choice for this experiment.

### 3.3. Preparation and Performance Analysis of Phase Change Microcapsule Infrared Camouflage Fabric

**Figure 6:**
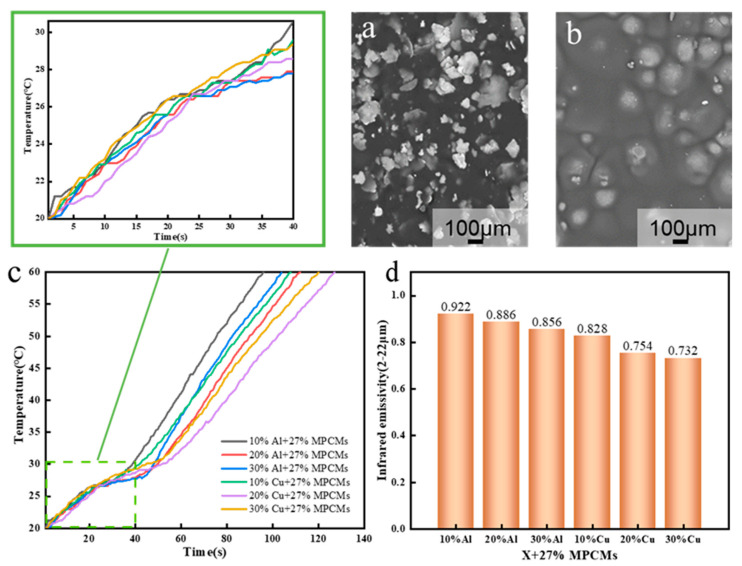
(**a**) SEM image of copper powder, (**b**) SEM image of aluminum powder, (**c**) Temperature rise curve of single-layer infrared camouflage coating samples after adding different kinds and contents of low emissivity materials, (**d**) Infrared emissivity of single layer infrared camouflage coating sample.

According to the principle and preparation process described in Section 2.2.4, the infrared camouflage fabric is prepared by combining temperature-regulating phase change microcapsules with low emissivity metal materials. The SEM of low emissivity metallic copper powder and aluminum powder is shown in Figure 6a,b. According to the experimental results in Section 3.2, the content of phase change microcapsule is 27% and the coating thickness is 1.5 mm. Figure 6c shows the temperature rise curve of single-layer infrared camouflage coating sample after adding different kinds and contents of low emissivity materials. Figure 6d shows the infrared emissivity of the sample.

By analyzing the heating curve of the sample, it can be seen that it takes a long time for the sample to rise from 20 to 60 °C when the copper powder is added to the phase change microcapsule solution, especially when the copper powder content is 20% and 30%. At the beginning of heating, when the content of copper powder is 20%, the heating rate of the sample is the lowest. When the aluminum powder is added to the phase change microcapsule solution, the temperature rise of the sample is relatively fast. The reason is that when the content of metal particles is the same, the spherical aluminum powder is isotropic, the particle size is small, the distribution is relatively uniform, and the surface morphology is regular, which makes it easier to form mutually contacted heat conduction network chains, so as to improve the heat conduction efficiency of the material. Therefore, the heat transfer of the aluminum powder sample is faster when heating up, which reduces the temperature control performance of the sample. The test results of the two groups of samples show that the temperature control performance of the samples is better when the addition amount of low emissivity material is 20%. As can be seen from Figure 6d, the infrared emissivity of the sample with copper powder is less than that of the sample with aluminum powder. The reason can be explained by the microscopic morphology of the two kinds of materials. It can be seen that the copper powder has a flaky structure (Figure 6a), and the aluminum powder has a spherical structure (Figure 6b). The particle thickness of the flaky structure is small, and at the same particle concentration (mass-specific gravity), the content of flaky particles in the coating is more [53]. The flake particles can be arranged horizontally in the coating to form a compact reflective layer. This arrangement can effectively reduce the emissivity of the coated fabric [54]. The higher the content of copper powder, the smaller the infrared emissivity of the sample, but when the content of copper powder is 20% and 30%, the emissivity of the sample is close. Considering the material cost and the flexibility of the coating, the content of copper powder can be determined as 20%.

**Figure 7:**
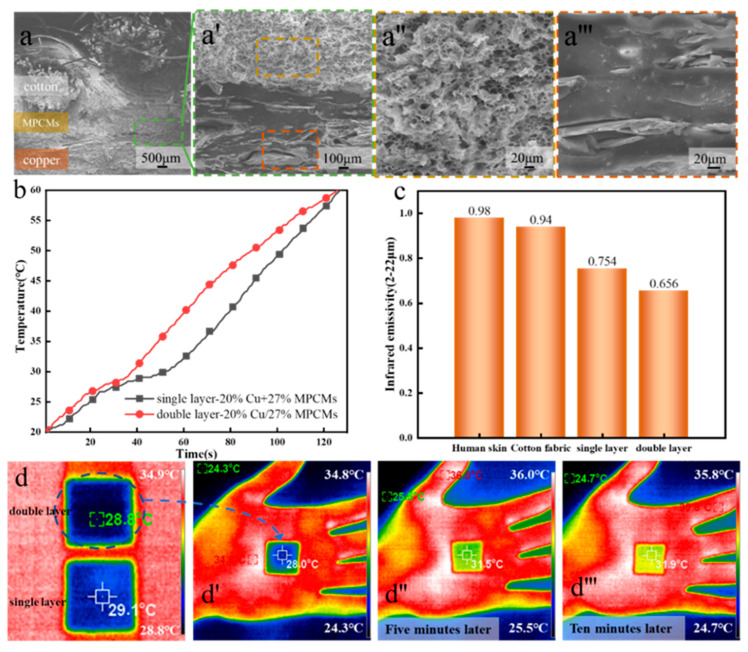
(**a**) Cross section SEM image of double-layer infrared camouflage fabric, (**b**) Comparison of temperature rise curves of single-layer and double-layer infrared camouflage fabrics, (**c**) Infrared emissivity of human skin, cotton fabric, single-layer and double-layer infrared camouflage fabrics, (**d**) Infrared thermogram of double-layer infrared camouflage fabric.

According to the theoretical analysis, two factors should be considered in infrared camouflage: temperature and infrared emissivity. It is considered that in this article, the infrared camouflage performance of the sample is the best when copper powder with low emissivity is added to the phase change microcapsule solution and the content of copper powder is 20%. At present, the prepared samples are all single-layer coatings. In order to ensure the ability of temperature regulation and reduce the infrared emissivity of the sample as much as possible, the double-layer coating can be prepared with copper powder and phase change microcapsule. The bottom layer is a phase change microcapsule (content: 27%) coating to ensure the temperature regulation performance of the sample, and the surface layer is a copper powder (content: 20%) coating to reduce the infrared emissivity of the sample (Figure 7a), while other factors remain unchanged.

According to the temperature rise data in Figure 7b, the time required for the double-layer and single-layer samples to rise from 20 to 60 °C is very close, that is, the two samples have the same temperature adjustment ability. The infrared emissivity of the double-layer sample is significantly lower than that of the single-layer sample (Figure 7c). Therefore, the infrared camouflage performance of the double-layer coating sample is better. This conclusion is also confirmed in the infrared thermogram (Figure 7d). The double-layer infrared camouflage fabric is close to the environment in the infrared thermal image, which can reduce the detected surface temperature of human skin by 6.8 °C, and can still reduce 3.9 °C after covering for 10 min.

### 3.4. Analysis of Mechanical Properties of Phase Change Microcapsule Infrared Camouflage Fabric

**Figure 8:**
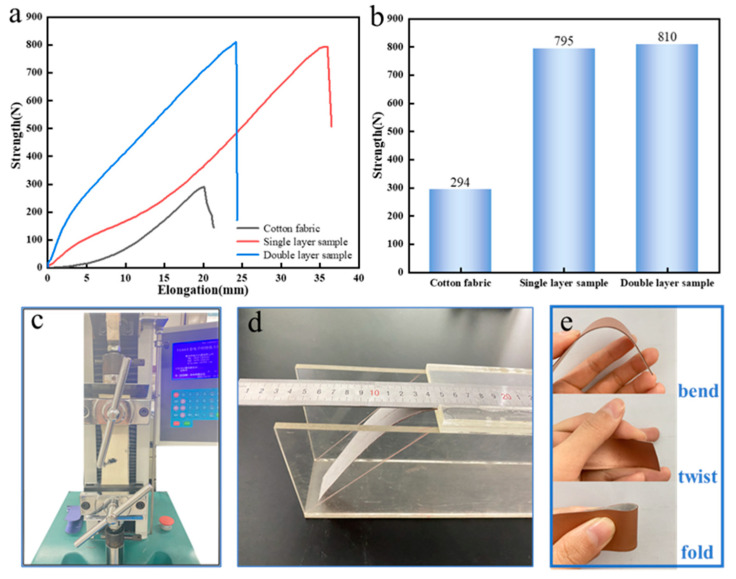
(**a**) Tensile strength displacement curve of infrared camouflage fabric, (**b**) Maximum tensile strength of infrared camouflage fabric, (**c**) Tensile strength test of infrared camouflage fabric, (**d**) Measurement of bending length of infrared camouflage fabric, (**e**) Soft performance display of infrared camouflage fabric.

The tensile and tear properties of infrared camouflage fabrics are tested by universal strength machine (Figure 8c), with reference to standard ISO34-1 [55]. The sample size is 200 × 50 mm, the clamping distance is 100 mm, and the tensile speed is 100 mm/min. Each sample shall be tested for 5 times, and the average value shall be taken as the final test result. The test results are shown in Figure 8a,b and Table 2. According to standard ISO 4604: 2011 [56], the coated fabric’s extension length was measured using a fixed angle bending machine (Figure 8d), and the bending stiffness G of the coated fabric was about 3.36 mN · m according to Formula (2). YG (B) 401E Martindale wear tester is used to test the wear resistance of infrared camouflage fabric, with reference to standard ISO12947-3 [57]. The diameter of the sample is 38 mm. Record the mass loss of the fabric when rubbing 100 times, 250 times, 500 times, 750 times, and 1000 times respectively. Calculate the wear resistance index according to Formula (3). The results are shown in Table 3.

*G* = 9.81*ρ_A_* (*L*/2) ^3^ (2)

where: *G* is the ordinary bending stiffness (mN · m), *ρ_A_* is the mass per unit area (g/m^2^), referring to standard ISO3374, and *L* is the average extension length (m).

*Ai* = *n*/∆*m* (3)

where *Ai* is the wear resistance index, the unit is times per milligram (times/mg); *n* is the total friction times, unit: times; ∆*m* is the mass loss of the sample under the total friction times, the unit is mg.

The test shows that the tensile and tear strength of infrared camouflage fabric is much higher than that of cotton fabric. The tensile strength of double-layer infrared camouflage fabric is the largest (810 N), but the tensile displacement is less than that of single-layer infrared camouflage fabric. The calculation results of the fabric wear index show that the wear indexes of the two infrared camouflage fabrics are similar, and the more times of wear, the better the wear resistance. According to the actual picture of the fabric (Figure 8e) and the bending length test of the material, the infrared camouflage fabric has good softness and crimp ability. Table 4 shows the performance comparison between this material and other infrared camouflage materials. The results show that the infrared camouflage fabric has good mechanical properties and softness.

## 4. Conclusions

Based on the principle of infrared camouflage and the characteristics of textile materials, from the point of view of controlling the temperature of materials to achieve the purpose of camouflage, phase change microcapsule temperature-regulating composite material with different parameters were prepared by padding method and coating method, and their infrared radiation performance was analyzed. The infrared camouflage textile composite was prepared by combining phase change microcapsule material with low emissivity metal material and using high molecular polyurethane as the matrix. The results of differential scanning calorimetry show that the phase change microcapsules melt and absorb heat at about 28 °C when heating up, and solidify and release heat at about 35 °C when cooling down. The temperature rises test shows that the content of adhesive, the content of phase change microcapsule, the thickness of the coating, and thermal conductivity have a great influence on the temperature adjustment ability of the sample. The temperature-regulating textile materials with phase change microcapsules were prepared by the coating method. When the content of phase change microcapsules is 27% and the coating thickness is 1.5 mm, the performance of the sample is the best. The results of the infrared thermogram and infrared emissivity test show that when the content of phase change microcapsule in the bottom layer is 27% and the content of flake copper powder in the surface layer is 20%, the double-layer coating sample has a good infrared camouflage effect. Its infrared emissivity in the band of 2–22 μm is 0.656, covering it on the surface of the human body can reduce the temperature to 6.8 °C, and effectively reduce the infrared radiation. Based on these results, we believe that this study can provide a reference for the preparation of infrared camouflage composite material in the laboratory and industry. The infrared camouflage textile composite is expected to be used in military textiles such as individual protective clothing, military tents, and equipment tarpaulins.

## Figures and Tables

**Table 1:** Material Information.

Material	Particle Size/Model	Manufacturer
Hollow glass beads	30–100 μm	Henan Bairun casting materials, China
Silicon dioxide	20 nm	Jiangsu Tian xing’s new materials, China
Copper powder	38 μm	Nangong Xindun alloy welding mate-rial spraying, China
Aluminum powder	25 μm	Shanghai Aladdin Bio-chemical Technology, China
Polyurethane	PU2540	Guangzhou Yuheng environmental protection materials, China
Defoamer	AFE-1410	Shandong Yousuo Chemical Technology, China
Thickener	7011	Guangzhou Dianmu Composite Materials Business Department, China
Dispersant	5040	Shandong Yousuo Chemical Technology, China
Urea, Formaldehyde aqueous solutions		Meryer (Shanghai) Chemical Technology, China
Paraffin, OP emulsifier, Triethanolamine, Citric acid, Petroleum ether		Beijing enokai Technology, China

**Table 2:** Tear strength of infrared camouflage fabric.

	Load (N)	Displacement (mm)	Tear Strength (N)
Cotton fabric	5.4	50.05	6.63
Single layer infrared camouflage fabric	7.5	50.05	21.31
Double layer infrared camouflage fabric	14.5	50.05	14.67

**Table 3:** Wear resistance index of infrared camouflage fabric with different friction times.

Friction Times	100	250	500	750	1000
Single layer infrared camouflage fabric	4.35	6.02	7.58	8.82	9.66
Double layer infrared camouflage fabric	3.85	4.90	6.71	7.77	8.77

**Table 4:** Performance comparison between this material and other infrared camouflage materials.

Infrared Camouflage Materials	Infrared Emissivity (8–14 μm)	Temperature Regulation Range (°C)	Tensile Strength (N)
This material	0.507 0.656 (2–22 μm)	6.8	810
Material 1 [45]	0.575	5–10	
Material 2 [23]	0.795		398.4

## Data Availability

The data that support the findings of this study are available within the article.
